# Lifestyle-Medikament Vitamin D. Was gibt es an Evidenz?

**DOI:** 10.1007/s00393-023-01392-9

**Published:** 2023-07-28

**Authors:** Uwe Lange, Nils Schulz, Philipp Klemm

**Affiliations:** grid.8664.c 0000 0001 2165 8627 Abt. Rheumatologie, klin. Immunologie, Osteologie und Physikalische Medizin, Campus Kerckhoff der Justus-Liebig-Universität Gießen, Benekestr. 2–8, 61231 Bad Nauheim, Deutschland

**Keywords:** Vitamin D, Biosynthese, Symptome bei Defizit, Evidenz zur Supplementation, Vitamin D, Biosynthesis, Symptoms of deficit, Evidence on supplementation

## Abstract

Eine Unterversorgung von 25(OH)Vitamin‑D_3_ (Calcidiol) besteht in vielen Ländern mit mäßiger Sonnenlichteinstrahlung, langen Wintern und nur mäßigem Fischkonsum. Risikogruppen für einen Vitamin‑D_3_-Mangel stellen ältere Personen über 65 Jahre dar, geriatrische Personen in Pflegeheimen, Säuglinge und Kinder/Jugendliche. Somit bestehen auch in Deutschland viele Situationen, welche eine Vitamin-D-Substitution rechtfertigen. Aktuell wird Vitamin‑D_3_ jedoch als „Wunderwaffe“ gegen alles angepriesen. Aber wie sieht die Datenlage aus? Wo kann es helfen und wo nicht?

## Abstract (en)

An undersupply of 25-(OH) vitamin D3 (calcifediol) exists in many countries with moderate sunlight, long winters and only moderate fish consumption. Risk groups for vitamin D3 deficiency are older persons over 65 years, geriatric persons in nursing homes, infants and children/adolescents. Therefore, there are also many situations in Germany which justify vitamin D substitution; however, vitamin D3 is currently praised as a “magic bullet” against everything. But what do the data look like? Where can it help and where can it not help?

## Biosynthese von Vitamin D

Das fettlösliche Vitamin D (Cholecalciferol) zählt zu den Secosteroiden und wird zu ca. 80–90 % in der Haut aus 7‑Dehydrocholesterol unter UV-B-Strahlung synthetisiert. Mit zunehmendem Alter sinkt der Gehalt an 7‑Dehydrocholesterol in der Haut und die Fähigkeit zur Vitamin-D-Bildung um den Faktor 3 bis 4 im Vergleich zu 20-jährigen Personen ab [1, 2]. 25(OH)Vitamin‑D_3_ (Calcidiol, im Folgenden Vitamin D_3_ genannt) ist die hepatische Speicherform des Vitamin D_3_, und seine Analyse dient dem Nachweis einer mittel- bis langfristigen Unterversorgung. Renal wird durch die 1α-Hydroxylase aus 25(OH)Vitamin‑D_3_ das biologisch aktive 1,25(OH)_2_-Vitamin‑D_3_ (Calcitriol, D‑Hormon) als aktivierter Ligand für den Vitamin-D-Rezeptor (VDR) gebildet bzw. durch die 24-Hydroxylase zum 24R,25(OH)_2_-Vitamin‑D_3_ inaktiviert. Die renale Bildung von 1,25(OH)_2_-Vitamin‑D_3_ wird direkt gefördert durch erhöhtes Parathormon (PTH), verringerte Kalziumspiegel und niedrige Phosphatspiegel. Indirekt (zumeist über das PTH) beeinflussen Kalzium, Östrogen, Glukokortikoide, Calcitonin, Somatotropin und Prolaktin die Bildung von 1,25(OH)_2_-Vitamin‑D_3_ [2]. In anderen Geweben induzieren u. a. Zytokine und Wachstumsfaktoren die Aktivierung von 25(OH)Vitamin‑D_3_ zu 1,25(OH)_2_-Vitamin‑D_3_ [3]. In den Zellen der Zielorgane wirkt 1,25(OH)_2_-Vitamin‑D_3_ wie ein Steroidhormon (Bindung an den intrazellulären VDR und Transport in den Zellkern, dort Einflussnahme auf die Transkription hormonsensibler Gene mit Änderungen der Proteinsynthese) [2].

## Funktion – Störungen – Mangel von Vitamin D

Vitamin D_3_ kommt eine zentrale Rolle bei der Regulierung des Kalziumhaushaltes und beim Knochenaufbau zu.

Bekanntermaßen führt ein Vitamin‑D_3_-Mangel bei Kindern zu einer Rachitis und bei Erwachsenen zu einer Osteomalazie. Auf der anderen Seite kann bei Tuberkulose, Sarkoidose und anderen granulomatösen Erkrankungen verstärkt, z. B. in Makrophagen, biologisch aktives 1,25(OH)_2_-Vitamin‑D_3_ aus 25(OH)Vitamin‑D_3_ gebildet werden mit konsekutiver Hyperkalzämie [4].

Zwischen diesen Extremen findet sich eine Grauzone. Die Definition eines Vitamin‑D_3_-Mangels ist unverändert kontrovers. Das amerikanische Institute of Medicine benennt als unteren Grenzwert 20 ng/ml [5].

Eine Metaanalyse von 2016 bei verschiedenen europäischen Ländern zeigte bei 13 % eine Unterversorgung auf (< 12 ng/ml im Jahresdurchschnitt) [6]. In Deutschland ergab die DEGS1-Erhebung in den Monaten November bis April in 25 % einen schweren Vitamin‑D_3_-Mangel und in den Monaten Februar und März bei mehr als 50 % [7].

Genetische Polymorphismen im Vitamin-D-Rezeptor(*VDR*)-Gen können die Expression oder Funktion des Rezeptors modulieren und damit konsekutiv die Aufnahme, die Prozessierung und Wirkung von Vitamin D_3_ verändern sowie auch auf verschiedene Immunprozesse haben. So sind potenzielle Zusammenhänge zwischen genetischen Variationen im Vitamin-D-Signalweg und Infektionen im Kleinkindalter bekannt [8]. Ebenso sind Beeinträchtigungen der Hormonempfindlichkeit und der Tumorprognose bei Mamma- und Lungentumoren beschrieben [9, 10]. Der Zusammenhang von VDR-Polymorphismen mit dem genetischen Risiko und klinischen Merkmalen wird bei Autoimmunerkrankungen wie der multiplen Sklerose (MS), rheumatoiden Arthritis (RA) und dem systemischen Lupus erythematodes (SLE) diskutiert [11]. Ebenso sind VDR-Polymorphismen mit Autoimmunerkrankungen des Bindegewebes und dem Risiko für die Entwicklung einer RA assoziiert [12, 13]. Zu erwähnen ist allerdings, dass verschiedene Polymorphismen in unterschiedlichen Populationen das Risiko für eine RA erhöhen oder verringern können. Eine Sekundäranalyse einer randomisierten klinischen Studie deutet darauf hin, dass bestimmte Isoformen des VDR besonders von einer Vitamin‑D_3_-Supplementation i. R. einer Prävention kolorektaler Adenome profitieren könnten [14]. An dieser Stelle sei auf eine Übersichtsarbeit, die den Einfluss des genetischen Hintergrundes für Vitamin D_3_ und andere Nahrungsbestandteile zusammenfassend schildert, verwiesen [15]. Anzumerken bleibt, dass die bisherige Studiendatenlage deutliche Heterogenitäten mit entsprechenden Limitationen zeigt.

Neben genetischen Faktoren hat auch der Body Mass Index (BMI) einen Einfluss auf die Vitamin‑D_3_-Resorption [1]. Dieser Aspekt wird meist bei der Interpretation von Studiendaten kaum berücksichtigt. Auch bei der VITAL-Studie bleibt unklar, ob das Gewicht in irgendeiner Weise Einfluss auf die Resultate nahm. Die Faktoren Übergewicht und genetische Grundlagen sollten zukünftig explizit bei der Interpretation von Studien berücksichtigt werden.

## Symptome bei Vitamin-D-Mangel

Die Symptome des Vitamin‑D_3_-Mangels sind unspezifisch, und es können diffuse Knochen- und Muskelschmerzen sowie Muskelschwäche und Frakturen auftreten [2]. Zudem sind Neigungen zur Tetanie, muskuläre Hypotonie, epileptische Anfälle, Herzrhythmusstörungen, erhöhte Infektanfälligkeit und eine Gingivahyperplasie beschrieben. Neben diesen Symptomen sind auch autokrine Funktionen des Vitamin-D-Systems bei der Zelldifferenzierung, Zellproliferation, Apoptose und Immunmodulation bekannt [16]. Somit steuert und moduliert Vitamin D_3_ das Immunsystem, wobei die genauen Zusammenhänge noch nicht völlig erforscht sind.

## Vitamin-D-Mangel als Risikofaktor

Demnach scheint Vitamin D_3_ eine wichtige, fast ubiquitär (essenzielle) Rolle im Körper zu spielen. Konkordant stellt nach aktueller Studienlage eine Unterversorgung mit Vitamin D_3_ möglicherweise einen Risikofaktor u. a. für MS, Diabetes mellitus Typ 1, SLE, Fibromyalgie, Tuberkulose und Atemwegsinfekte dar [17–22]. Bei älteren Personen scheint ein Vitamin‑D_3_-Defizit ein Risikofaktor für Osteoporose [23–25], Morbus Parkinson [18, 23], Schlafapnoesyndrom [26] und Tagesmüdigkeit [26, 27] darzustellen.

## Evidenz zur Vitamin-D_3_-Supplementation

### Herz-Kreislauf-System

Im Jahr 2019 wurde die bisher größte und längste randomisierte placebokontrollierte US-amerikanische Interventionsstudie (VITAL-Studie) veröffentlicht. Durch eine Omega-3-Fettsäuren- und Vitamin‑D_3_-Supplementation konnte das Risiko im Sinne einer Primärprävention von malignen Erkrankungen oder schweren kardiovaskulären Ereignissen (Herzinfarkt, Schlaganfall oder kardiovaskulärer Tod) bei Männern > 50 Jahren und Frauen > 55 Jahren nicht gesenkt werden [28]. Eine Metaanalyse von 2019 sowie eine weitere Studie konnten ebenfalls durch eine Vitamin‑D_3_-Supplementation keine Herz-Kreislauf-Protektion detektieren [29, 30].

Eine im verblindeten RCT-Design durchgeführte finnische Studie aus dem Jahr 2022 konnte keinen Einfluss einer Vitamin‑D_3_-Substitution mit 1600 IU/Tag oder 3200 IU/Tag auf die Entstehung von kardiovaskulären Erkrankungen oder Malignomen bei 2495 Männern über 60 Jahre und Frauen über 65 Jahre im Vergleich zu Placebo über einen Zeittraum von 5 Jahren detektieren [31].

Anzumerken ist, dass es sich bei der VITAL- wie auch der finnischen Studie um Primärinterventionsstudien handelt. Ein Ereignis soll durch die Intervention nicht eintreten. Es werden relativ gesunde Probanden aus der Gesamtbevölkerung rekrutiert. Alleinig das gewählte Alter war ein Risikofaktor. Somit sind die Daten nur zum Teil auf Risikokollektive und/oder zur sekundären Prophylaxe übertragbar. Wenn man sich jedoch persönlich mit den rekrutierten Probanden vergleichen/identifizieren kann, sind die Ergebnisse und die Ableitung klar: Eine Vitamin‑D_3_-Substitution schützt nicht vor kardiovaskulären Erkrankungen.

### Autoimmunerkrankungen

Vitamin D_3_ wird unverändert als ein Modulator des Immunsystems angesehen [32, 33].

Die Frage, ob eine Vitamin‑D_3_- oder eine Omega-3-Fettsäure-Substitution autoimmune Erkrankungen verhindern kann, wurde in einer zusätzlichen Untersuchung der VITAL-Studie verfolgt [34]. Primärer Endpunkt dieser Untersuchung war das Auftreten einer autoimmunen Erkrankung (u. a. RA, Polymyalgia rheumatica, autoimmune Schilddrüsenerkrankungen, Psoriasis vulgaris, chronisch entzündliche Darmerkrankungen). Es zeigte sich insgesamt ein reduziertes Auftreten von Autoimmunerkrankungen von 22 % im Vitamin‑D_3_-Arm und von 15 % im Omega-3-Fettsäuren-Arm Vergleich zu Placebo, wobei das prädefinierte Signifikanzniveau nicht erreicht wurde. Korrespondierend zeigte sich in einer laborchemischen Zusatzuntersuchung der VITAL-Studie eine Reduktion vom hoch-sensitiven CRP, nicht jedoch von Interleukin(IL)‑6, -10 oder Tumor-Nekrose-Faktor‑α im Vitamin‑D_3_-Arm nach 2, jedoch nicht 4 Jahren Substitution. Keine signifikanten Veränderungen waren unter Substitution von Omega‑3-Fettsäuren eruierbar [35].

Interessant sind aktuelle 7‑Jahres-Daten, die nach Beendigung einer Vitamin‑D_3_-Supplementation mit 2000 IE/Tag über den Zeitraum von 5,3 Jahren eine signifikant reduzierte Inzidenz von Autoimmunerkrankungen aufzeigen, allerdings waren die protektiven Effekte 2 Jahre nach Beendigung der Supplementation nicht mehr nachweisbar, was für eine kontinuierliche Vitamin‑D_3_-Einnahme sprechen würde [36].

In einer Metaanalyse ergaben sich Hinweise, dass eine Vitamin‑D_3_-Supplementation das Risiko einer Exazerbation einer RA reduzieren kann [37]. Einer weiteren Metaanalyse der EULAR-Taskforce ist zu entnehmen, dass hochrangige Belege für klinisch bedeutsame Wirkungen einzelner Nahrungsmittelbestandteile einschließlich Vitamin D_3_ auf den Krankheitsverlauf fehlen [38].

### Frailty – Stürze – Frakturverhinderung

Bei Risikopersonen konnten durch eine adäquate Supplementation sowohl Stürze und Frakturen verhindert werden [23–25].

Basierend auf den Daten der VITAL-Studie wurde der Einfluss einer Vitamin‑D_3_-Substitution auf Gebrechlichkeit (Frailty) [39] und Frakturen [40] untersucht. Weder eine Vitamin‑D_3_- noch eine Omega-3-Fettsäuren-Substitution hatten einen signifikanten Einfluss auf den Gebrechlichkeitsindex [39]. Auch die Inzidenz von Gebrechlichkeit zeigte sich nicht signifikant beeinflusst. Insgesamt scheint eine Supplementation von Vitamin D_3_ und Omega-3-Fettsäuren nicht zur primären Prophylaxe von Gebrechlichkeit bei älteren, generell gesunden Menschen ohne Vitamin‑D_3_-Mangel geeignet.

In einer weiteren Analyse wurde daher der Einfluss der Vitamin‑D_3_- und/oder Omega-3-Fettsäuren-Einnahme auf das Frakturrisiko untersucht [40]. Vorab konnte durch die gleiche Arbeitsgruppe, basierend auf den VITAL-Daten, gezeigt werden, dass eine Vitamin‑D_3_-Einnahme keinen Effekt auf die axiale oder periphere Knochendichte (gemessen mittels dualer Photonenabsorptionsmetrie [DXA]) und volumetrische Knochendichte an Radius und Tibia (gemessen mittels peripherer quantitativer CT) hatte [41].

Es zeigte sich somit kein Effekt einer Vitamin‑D_3_-Substitution auf das Frakturrisiko bei älteren, gesunden Menschen, welche nicht speziell für einen Vitamin‑D_3_-Mangel, geringe Knochendichte und Osteoporose ausgewählt wurden.

Es muss nochmals darauf hingewiesen werden, dass die VITAL-Studie nicht „Knochenkranke und Gebrechliche“ rekrutierte, sondern v. a. „Best-Agers“ ohne nennenswerte Probleme. Dennoch ist die Aussagekraft für diese Kohorte aufgrund des Designs und der Probandenzahl valide: Vitamin D_3_ verhindert bei „Best-Agern“ weder Frakturen noch Gebrechlichkeit. Vitamin D_3_ hat in der Population auch keine Auswirkung auf die Knochendichte (DXA, QCT).

### Ateminfekte und COVID-19-Infektion

Bei Kindern und Jugendlichen (1 bis 16 Jahre) ist durch eine Vitamin‑D_3_-Supplementation von 400–1000 IE/Tag ein schwacher Schutzeffekt vor akuten Ateminfektionen bekannt, wobei höhere Dosierungen keinen Vorteil ergaben [42, 43].

In einer kleineren 2022 publizierten doppelt-verblindeten randomisierten kontrollierten Studie mit 192 analysierten Probanden konnte ein signifikant protektiver Effekt einer Vitamin‑D_3_-Supplementation mit 4000 IU/Tag gegenüber einer SARS-CoV-2-Infektion (relatives Risiko [RR] 0,23; 95 %-CI: 0,09–0,55) detektiert werden [44]. In kritischer Begutachtung der Resultate liegen jedoch mögliche Konfundierungseffekte vor: Sowohl COVID-19 wie auch Vitamin‑D_3_-Defizienz sind unabhängig mit verschiedenen Risikofaktoren bzw. Störfaktoren wie Übergewicht, höherem Lebensalter (> 65 Jahre) und männlichem Geschlecht assoziiert [45]. Somit kann bzw. konnte bislang ein protektiver Effekt einer Vitamin‑D_3_-Substitution bezüglich einer COVID-19-Infektion wie auch gegenüber Infektionen der oberen Atemwege nicht klar beantwortet werden.

Zwei aktuelle große europäische randomisierte kontrollierte Studien (CLOC und CORONAVIT) zeigten keinen Benefit einer Vitamin‑D_3_-Supplemenation bezüglich akuter respiratorischer Effekte und einer COVID-19-Infektion bei Erwachsenen [46, 47]. Sowohl das National Institutes of Health und das britische National Institute of Health and Care Excellence sehen keine Evidenz für oder gegen eine Vitamin‑D_3_-Gabe, um COVID-19 zu behandeln oder davor zu schützen [48, 49]. Zum gleichen Resultat kommen ein Cochrane Metareview und das Robert Koch-Institut [50, 51].

Ein systematischer Review vom November 2020 zeigte, dass ein Vitamin‑D_3_-Mangel nicht mit erhöhter Infektanfälligkeit von COVID-19 assoziiert ist. Es zeigte sich aber eine positive Korrelation zwischen Vitamin‑D_3_-Mangel und der Schwere einer COVID-Infektion inklusive Todesfälle [50]. Das akute Lungenversagen stellt eine schwerwiegende Komplikation bei COVID-19-Infektion dar, es kann durch einen Vitamin‑D_3_-Mangel verschlimmert werden [51].

Abschließend bleibt festzuhalten, dass eine Vitamin‑D_3_-Substitution in Unwissenheit des Vitamin‑D_3_-Spiegels nicht protektiv gegenüber einer SARS-CoV-2- oder andersartigen Infektionen der oberen Atemwege ist und somit (aktuell) nicht für die Praxis empfohlen werden kann. Auch bei bekanntem Vitamin‑D_3_-Mangel ist bei Gesunden keine protektive Wirkung einer Supplementation zu erwarten. Inwiefern es in Risikokollektiven für einen Vitamin‑D_3_-Mangel und bei Infektionen (Stichwort: geriatrischer Patient/In im Pflegeheim) aussieht, ist jedoch nicht geklärt und eine individuelle Entscheidung.

### Malignome

Eine Metaanalyse von 2014 konnte aufzeigen, dass ein Vitamin‑D_3_-Mangel wahrscheinlich nicht mit einem erhöhten Malignomrisiko einhergeht, aber gleichwohl bei bestehender maligner Erkrankung sich negativ auf den Verlauf auswirken könnte [40]. Bestätigt wurden 2017 die Resultate des wahrscheinlich nicht vorhandenen Einflusses auf die Malignomentstehung bei 7 differenten Malignomen anhand genetischer und epidemiologischer Daten [52–54]. Auch das RKI sieht keinen kausalen Zusammenhang für Krebserkrankungen.

Eine Metaanalyse klinischer Studien von 2019 belegt unter Vitamin‑D_3_-Supplementierung i. R. einer Antikrebstherapie eine Verringerung der Sterberate um etwa 13 %, jedoch keine Verminderung des Malignomrisikos [55–57]. Bei der Datenübertragung durch das Deutsche Krebsforschungsinstitut im Jahr 2021 auf Deutschland wurde errechnet, dass eine Vitamin‑D_3_-Supplementierung aller Deutschen > 50 Jahre möglicherweise bis zu 30.000 Todesfälle durch maligne Erkrankungen verhindern könnte, aber keineswegs eine spezifische Antikrebstherapie ersetzt [58]. Auch die Sterberate bei Lungenerkrankungen scheint durch eine adäquate Vitamin‑D_3_-Versorgung positiv beeinflusst zu werden [43].

In der VITAL-Studie konnte durch eine Vitamin‑D_3_-Supplementation das Risiko für maligne Erkrankungen nicht gesenkt werden, auch für die Prävention erwies sich die Supplementation ungeeignet [28].

## Fazit für die Praxis

Insgesamt scheint die Datenlage die hohe öffentliche Meinung einer Vitamin‑D_3_-Supplementation als „Wunderwaffe“ nicht zu rechtfertigen. Auch die brandaktuelle Leitlinie für den Lebensstil bei entzündlich rheumatischen Erkrankungen belegt keine Evidenz für Nahrungssupplemente. Der gezielte Einsatz kann in einzelnen Indikationen bei vorliegendem Vitamin‑D_3_-Mangel jedoch sinnvoll sein.
